# Referral and admission to intensive care: A qualitative study of doctors’ practices in a Tanzanian university hospital

**DOI:** 10.1371/journal.pone.0224355

**Published:** 2019-10-29

**Authors:** Sofia Engdahl Mtango, Edwin Lugazia, Ulrika Baker, Yvonne Johansson, Tim Baker

**Affiliations:** 1 Department of Acute Internal Medicine and Geriatrics in Linköping, and Department of Medical and Health Sciences, Linköping University, Linköping, Sweden; 2 Department of Anaesthesia & Intensive Care, Muhimbili University of Health and Allied Sciences, Dar Es Salaam, Tanzania; 3 College of Medicine, University of Malawi, Blantyre, Malawi; 4 Department of Public Health Sciences, Karolinska Institute, Stockholm, Sweden; University of Heidelberg, GERMANY

## Abstract

**Background:**

Intensive care is care for critically ill patients with potentially reversible conditions. Patient selection for intensive care should be based on potential benefit but since demand exceeds availability, rationing is needed. In Tanzania, the availability of Intensive Care Units (ICUs) is very limited and the practices for selecting patients for intensive care are not known. The aim of this study was to explore doctors’ experiences and perceptions of ICU referral and admission processes in a university hospital in Tanzania.

**Methods:**

We performed a qualitative study using semi-structured interviews with fifteen doctors involved in the recent care of critically ill patients in university hospital in Tanzania. Inductive conventional content analysis was applied for the analysis of interview notes to derive categories and sub-categories.

**Results:**

Two main categories were identified, (i) difficulties with the identification of critically ill patients in the wards and (ii) a lack of structured triaging to the ICU. A lack of critical care knowledge and communication barriers were described as preventing identification of critically ill patients. Triaging to the ICU was affected by a lack of guidelines for admission, diverging ideas about ICU indications and contraindications, the lack of bed capacity in the ICU and non-medical factors such as a fear of repercussions.

**Conclusion:**

Critically ill patients may not be identified in general wards in a Tanzanian university hospital and the triaging process for the admission of patients to intensive care is convoluted and not explicit. The findings indicate a potential for improved patient selection that could optimize the use of scarce ICU resources, leading to better patient outcomes.

## Introduction

Intensive care is in-hospital care for patients with potentially reversible critical conditions that can benefit from closer monitoring and more invasive treatments than general wards can provide [1–3]. Demand for intensive care often exceeds availability, even in High-Income Countries (HICs), and rationing access to intensive care is therefore needed [4–12]. Rationing has been described as “the allocation of healthcare resources in the face of limited availability, which necessarily means that beneficial interventions are withheld from some individuals” [13] and is argued to be necessary to ensure ethical allocation of medical services [13]. In studies of Intensive Care Unit (ICU) admissions in situations of limited ICU space or resources, HIC triaging physicians have responded by using the resources more efficiently, for example by restricting ICU admission to the patients perceived to benefit most and transferring patients out of ICU sooner [6, 7]. According the World Federation of Societies of Intensive and Critical Care Medicine patients who are most likely to benefit from intensive care should be prioritized and ICU admission criteria should be developed to identify those patients [14]. Clear admission guidelines may be valuable in decreasing the impact of bias and prejudice [15] and limiting unnecessary use of resources [16] even though guidelines might not always be strictly followed [12, 15].

Decisions about patient “selection” for intensive care—ie which patients to refer and admit to intensive care—is complex and ethically challenging. The threshold for admission varies with available resources [17] and between hospitals [18]. In addition, physicians’ perceptions and predictions about the benefit of intensive care vary [19, 20]. Timely assessment of potential benefits and risks for the patients should be the first step in the selection process [1], and patients suitable for intensive care should be identified and admitted before their condition progresses beyond possible recovery [2].

Most research on patient selection for intensive care comes from HICs. Patient diagnosis, illness severity, surgical status, age and number of available beds have been found to impact physicians’ triage decisions [9, 11]. Bias and other non-medical factors such as triaging physician seniority, interprofessional conflicts and family demands for life support also affect decision making [8, 10, 21, 22]. Little is known about patient selection for intensive care in low income countries (LICs). While the burden of critical illness is higher in LICs than in HICs, there are far fewer ICU beds [23]. Uganda and Malawi, for example, have only one ICU bed per one million population [24, 25]. Given the huge discrepancy between the need and the resources for intensive care, rationing and the associated ethical issues are of particular importance [25].

Tanzania is a LIC in East Africa with a population of 57 million [26]. Availability of Intensive Care Units is very limited—none of seven district hospitals surveyed in 2009 had ICUs and the four national referral hospitals had a total of only 38 ICU beds [27, 28]. In-ICU mortality in these four hospitals was estimated at 41% [28]. There are no national admission guidelines for intensive care in Tanzania [27]. The aim of this study was to understand doctors’ experiences and perceptions of ICU referral and admission processes in Muhimbili National Hospital (MNH), a University Hospital in Tanzania.

## Method

### Study design

We performed a qualitative study using inductive conventional content analysis of semi-structured interviews with doctors involved in the recent care of critically ill patients.

### Study setting

Muhimbili National Hospital (MNH) in Dar es Salaam is Tanzania’s biggest referral hospital and serves as a University Teaching Hospital as well as the National Referral Hospital, receiving patients from all hospitals in the country. The hospital has 1300 beds. At the time of the study, the main ICU in MNH had six beds and admitted critically ill patients from the emergency room (18%), operating theatres (59%), wards (20%) and other hospitals (3%) [29]. One quarter (26%) were planned postoperative elective surgery admissions, 42% were admissions after emergency surgery and 32% were medical patients. The majority of patients were admitted for respiratory failure with 88% requiring oxygen therapy, 75% were intubated and 51% required mechanical ventilation (some patients were breathing through an endotracheal-tube without respiratory support) [30]. Monitoring on the ICU consisted of electrocardiography, non-invasive blood pressure and pulse oximetry. IV crystalloids, antibiotics, adrenaline and dopamine were available but noradrenaline, other inotropes, dialysis or continuous sedation were not. Less than 1% of the beds in MNH are assigned for intensive care [28], compared to for example 13% in the USA [31] and 7% in Cape Town, South Africa [16]. The in-hospital mortality rate of ICU patients is 50%.

The number of critically ill patients in MNH is not known. In a large government hospital in Uganda 12% of the patients were severely ill (MEWS ≥5) [32]. If MNH is similar to the population in the Ugandan study, then 143 patients are critically ill in MNH. With only 6 beds in the ICU, it would only be possible to admit 4% of those patients to ICU.

### Data collection

#### Participants

Individual interviews were conducted with doctors at MNH in June 2014. The main criterium for inclusion was a doctor who had been recently involved in the care of a critically ill patient, as identified in the ICU and the general wards by one of the researchers (SEM). This method of inclusion was chosen to gather data from doctors from various departments and with different levels of experience. An additional two ICU doctors who were not involved in any of the patient cases were interviewed. Four patient cases were selected purposively in a three-week period to get a mix of specialties and conditions: two in the ICU and two in general wards. Fifteen doctors were identified, aged between 25 and 60, from ICU and four general wards, and all agreed to participate. Participant characteristics can be seen in Table 1.

**Table 1:** Participant characteristics.

Participants	Number
Total	15
**Criterium for inclusion**	
Involved in one of the cases	13
ICU doctor	2
**Place of work**	
ICU	7
General Ward	8
**Cadre**	
Specialist	7
Resident (undergoing specialization)	7
Registrar (not yet undergoing specialization)	1
**Sex**	
Female	10
Male	5

#### Interviews

The interview guide (S1 Appendix) was structured around topic areas reflecting factors that may affect patient selection and prioritization processes for intensive care, as identified in previous studies. [8–10, 21, 22] These topic areas were: (1) knowledge of official ICU referral and admission criteria; (2) experiences of ICU referral and admission process and decision making in the specific patient case; (3) experiences of communication in the process of referral and admission to the ICU in general; (4) perceptions about indications for ICU care; (5) perceptions of the quality of care of critically ill patients in ICU and the general wards; (6) perceptions of influences of non-medical factors (such as “VIP patients” and patient fees) on patient selection for intensive care. The patient cases were used to open the interviews, enabling discussion about a real situation and making it easier to discuss sensitive and general issues. The interview guide was developed by the research team to contain few and open-ended questions, allowing free development of the content in the interviews. The interviewer also used open-ended and specific follow-up questions, such as asking the respondents to develop their answers. The questions were tested before use with staff in the hospital.

The interviewing researcher was a Swedish medical doctor (SEM) who had just completed a three months’ clinical attachment in MNH and was therefore known by most of the respondents. Working for several years with acute medicine and critical care, she had an understanding of the general underlying factors for critical care triage and had been involved in decisions about referral and admission to intensive care. While she had gained some knowledge about practices at MNH from her clinical attachments and had some cultural understanding from previous time spent in Tanzania, she was likely seen as an “outsider” by the respondents. The lead researcher’s position as a foreigner in this setting, with its potential implications for data collection, analysis and results, is outlined further in the discussion section.

To enable the project to be successfully conducted given the interviewees busy schedules, we made the decision to let the respondents decide freely where to be interviewed. Five interviews were conducted in the ICU, three in other wards and seven in an office or coffee room. Six interviews were done in private while the other nine were done in staff areas. The interviews conducted in staff areas were all done away from other people so that the conversations were not overheard. Interviews lasted between 15 minutes and one hour and all were conducted in English, the working language in the hospital. Notes were taken during the course of the interviews and detailed interview content, as remembered, was written down immediately afterwards. To make the interviews feasible in a busy hospital environment, and to encourage respondents to speak freely despite the sensitivity of some of the areas, no recordings were done.

### Analysis

Inductive conventional content analysis as described by Hsieh and Shannon [33] was applied for the analysis of interview notes. The interview notes were anonymized and numbered 1–15 and then read repeatedly by the first author (SEM) and one of the co-authors (YJ) to get familiar with the material and get a full picture of what was discussed during the interviews. Line-by-line coding was performed for each interview through condensing contents into meaning units which were labelled with preliminary codes. YJ coded three interviews independently to ensure consistent interpretation. Additional notes containing reflections and interpretations were taken during the coding process. Categories and sub-categories were developed from the codes, continually reassessed and adjusted, and some of the sub-categories with similar content were merged. The authors would revert back to the original interview notes in cases where a phrase was difficult to categorize, in order to reposition it in the context of the whole interview. The data were initially analysed separately for the ICU doctors and general ward doctors, however in the analysis the same categories appeared in both groups. Therefore, we made a methodological choice to analyse the interviews with the two groups of doctors together which we believe increased the richness of the analysis. The doctor categories (ICU and ward doctors respectively) were marked with different colours throughout the analysis making it possible to distinguish the answers and identify any differences between the two groups. The process of sorting categories and sub-categories was repeatedly done by both SEM and YJ, to ensure validity of the analysis [33]. Categories and subcategories were then organized into a hierarchical structure. Result interpretation and structuring were further supported by UB.

### Ethical considerations

Verbal informed consent was secured from all participants and was documented in the interviewer’s notes before the interviews started. The interviewer explained the purpose of the interview and that their participation would remain confidential. The names of the respondents were not shared with other staff in the hospital and were removed before analysis. According to our experience in the study setting there may have been cultural and political concerns with signing contract-like documents which may have limited participation. For this reason, our protocol that was approved by the ethics committee stated that the interviews will be anonymised and informed consent will be sought from the staff involved, but did not require written consent. As part of a large research collaboration, ethical clearance was granted by the National Institute for Medical Research in Tanzania (NIMR/HQ/R.8a/Vol.IX/1606), Muhimbili University of Health & Allied Sciences (MU/DRP/ AEC/Vol.XVI/125) and the Ethical Review Board in Stockholm (EPN/2015/673-31/2). Permission for the study was granted by The Tanzanian Commission for Science and Technology (2013-334-NA-2013-175) and by the Director of Medical Services at MNH (515 2013/2014).

## Results

Two main categories were identified: (1) identifying critically ill patients in the wards and (2) triaging patients to the intensive care unit (Table 2).

**Table 2:** Categories and sub-categories.

Category	Sub-category
**Identifying critically ill patients in the wards**	Knowledge and morale among staff
	Hierarchy and communication among staff
**Triaging patients to the intensive care unit**	Lack of space in ICU
	Material and human resources in the clinics
	Perceived indications and contraindications for intensive care
	Perceptions of expected benefit of intensive care
	Fear of repercussions
	VIP patients

### Identifying critically ill patients in the wards

Obstacles were described for the identification of critically ill patients in the wards and two subcategories were identified: (1) knowledge and morale among staff and (2) hierarchy and communication among staff.

#### Knowledge and morale among staff

Doctors perceived that critically ill patients do not get prioritized or even identified in the wards, sometimes due to the presence of so many critically ill and dying patients:

*“So we never even think about the main ICU*. *Because you know we kind of got so used to all these sick and dying patients”*

(no 12)

Doctors from both general wards and the ICU shared the view that there is a lack of knowledge about critical illness among the staff in the wards, especially among nurses but also doctors. Some nurses were described to lack training and knowledge, instead relying only on their prior experience with critically ill patients. At the same time it was mentioned as a problem that many nurses are new and lack experience as well.

*“…the nurses*’ *knowledge is bad*, *they don*’*t know what is an emergency case*. *The patient has to be really really sick for someone to notice*.*”*

(no 10)

It was not only a lack of knowledge that was seen as problematic. Doctors also described a low working morale among the nurses in the wards and a lack of drive to identify and care for the critically ill patients. The low working morale was, at least partly seen as a consequence of understaffing.

*“…because of being too few they just make up the vital signs*, *they write down the same vitals for all the patients*.*”*

(no 4)

Nurses were described as insecure, not taking initiatives or making decisions.

*“…they don*’*t dare to take any initiatives themselves and I think often they don*’*t believe themselves of being capable of doing things*.*”*

(no 5)

*“They are just sitting there not monitoring patients*, *they don*’*t even know how sick they are*. *Patients die here because of negligence and lack of responsibility*.*”*

(no 7)

#### Hierarchy and communication among staff

Hierarchy and its effects on communication between staff was mentioned as an obstacle to good quality care and identification of the critically ill patients.

The medical system with several hierarchical levels of professionals was described as creating a distance between the patients and the physician-in-charge. Since communication involves so many steps between the nurse who is closest to the patient and the specialist doctor, the information about the patient’s critical condition might not reach the specialist in time.

*“There*’*s a gap between the patients and the doctor*. *You know the system has so many levels; nurses -intern—registrar-resident-specialist*. *So the specialist doesn*’*t really know what*’*s going on with the patient*.*”*

(no 3)

The respondents described a routine in the hospital whereby contact between departments is done between staff of the same professional level. Referral to ICU is normally done between specialists—junior doctors may not be allowed. It becomes a time-consuming, difficult procedure to refer a patient to the ICU when a specialist is not available in the ward. According to the respondents specialists often also work in private clinics and are therefore absent from the hospital, even if they could still be reached by phone.

*“It is very hard for us to transfer a patient to the ICU*. *It has to be a specialist contacting the specialist on call in the ICU and often when we call our specialist about a sick patient they are busy with their own clinic”*

(no 8)

### Triaging patients to the intensive care unit

Factors were identified that affected the patient selection and prioritizing for ICU admission: factors that appeared to influence how the doctors reasoned about which patients get admitted or should be cared for in the ICU. Six subcategories concerning patient selection were identified: 1) lack of space in ICU, (2) material and human resources in the clinics, (3) perceived indications and contraindications for intensive care, (4) perceptions of expected benefit of intensive care, (5) fear of repercussions and (6) VIP patients.

#### Lack of space in ICU

Space in ICU was perceived as a significant problem, affecting the selection of patients. Ward doctors expressed that their ICU referrals were often turned down because of lack of space and that it could be pointless to contact ICU since it would be full. As a consequence, most patients were never considered for ICU. ICU was perceived to be more of a “last resort” as the few beds serve the entire hospital and patients need to be extremely ill to “qualify” for ICU referral.

*“The problem is the ICU is too small*, *it*’*s always full*. *[…] I think we*, *us doctors in the wards we totally lowered our criteria for how sick a patient needs to be to need ICU*, *like we think a patient needs to be really*, *really sick to go to the ICU*. *So we never even think about the main ICU*.*”*

(no 12)

The ICU beds were used for both acutely ill patients and patients after elective surgery. ICU doctors expressed that because of a lack of space, patients need to be strictly prioritized and may get declined admission even if they are perceived to be critically ill and potentially benefitting from ICU care. They also reasoned that because of the limited space, ICU cannot take patients that may need to stay for a long time.

*“We often have to say no because of lack of space*. *Those who are lucky come here*. *[…] Even if a patient qualifies for ICU you might have to say no because the beds are full because of for example elective dental cases*.*”*

(no 3)

#### Material and human resources in the clinics

The respondents described a lack of resources on the general wards. The deficiencies of supplies, diagnostic equipment, drugs, oxygen and staffing create a need for referral to ICU:

*“Some of them who need ICU could actually be managed here if we had better resources*, *for example this child who needed oxygen […] I sometimes even bring my own drugs you know*, *because I can*’*t stand it not giving the treatment they need*.*”*

(no 6)

The ward doctors expressed a belief that the quality of care in ICU is generally good and believed that a lot of patients can benefit from ICU care. Good nursing care was especially mentioned but also access to monitoring, oxygen and faster lab results than in the wards. However, a lack of drugs and equipment and the lack of an ICU doctor on call within the building, were perceived as reasons to keep critically ill patients on the general wards.

*“…we usually keep critically ill patients at our ward as long as possible*, *partly because then they are seen regularly and often … while at the ICU they risk to be forgotten*, *or they are not seen as often as when they are in our ward and the communication between the anesthesiologists and other specialists is very bad*.*”*

(no 1)

#### Perceived indications and contraindications for intensive care

Some of the ICU doctors had heard about MNH guidelines for ICU admission but were not using them. The interviewed doctors from the wards did not know about any ICU admission or referral guidelines.

Doctors expressed that post-operative observations and a need for mechanical ventilation are the main indications for ICU care. ICU doctors pointed out that it was a question of responsibility to keep the beds with respirators for patients who actually need a respirator. There was a notion among ward doctors that there is no point in contacting ICU about other types of patients.

*“the main ICU it*’*s exclusively for those who need ventilation … sending a patient to the ICU without the need for ventilation is also a way of backing off responsibility”*.

(no 7)

The need for close monitoring was also mentioned as a reason to be selected for ICU, because of better lab access, close surveillance from nurses and access to monitors, as well as need for oxygen therapy, which was more accessible in ICU than on the general wards.

Both ward and ICU doctors pointed out that there are differences between how the ICU doctors assess patients and that it is the personal judgement of the ICU doctor that controls which patients get admitted to the ICU.

*“It depends on who you will find there*, *some would always say no*, *some accept patients more easy*.*”*

(no 10)

When asked if there are certain conditions that should never be cared for in the ICU, (i.e. contraindications to ICU admission), different opinions were expressed among ward and ICU doctors. There was an idea that rules exist about not admitting contagious patients but ICU doctors expressed that it is difficult to say no if there is an available bed and the patient requires mechanical ventilation. HIV, TB and hepatitis were mentioned both as contraindications and as acceptable for care in the ICU.

#### Perceptions of expected benefit of intensive care

Potential benefit was mentioned as an important consideration when selecting patients for ICU care. Patients in shock (for example hypovolemic or septic shock) or who require only close monitoring and oxygen were seen as examples of patients who would not benefit more from ICU than care in the wards.

*“Shock patients should not be in the ICU*, *they can be managed in the wards*. *It*’*s easy*, *just give fluid*. *If you bring such patients to the ICU and if someone needs a ventilator*, *what do you do? You need to use your resources for those who most need it*.*”*

(no 7)

Poor prognosis was mentioned among both ICU and ward doctors, the patients admitted to the ICU should have a reasonable possibility of benefiting from ICU care. There was a conception that patients need to be prioritized to give ICU space for the ones who really need it. Unconscious patients, patients with renal failure and patients presenting to hospital too late were mentioned as examples of patients who don’t benefit from ICU care since there is nothing that can be done in ICU to save them.

*“I want to see patients that can really benefit*. *It*’*s painful to say no to patients without [a reasonably good] prognosis but sometimes you have to*.*”*

(no 15)

There was a perception among ward doctors that ICU do not want patients with infectious diseases like HIV, meningitis or tetanus because of poor prognosis or the risk that they will occupy the bed for too long.

*”They just say for example*—*oh you know this HIV patient with meningitis is going to die anyway so there is no point of bringing him*. *If you bring him it*’*s just a waste of time…*. *So basically we never call the ICU because we know they will say no anyway*.*”*

(no 8)

Also, legal aspects of withdrawal of futile care were brought up, a patient who is intubated cannot be extubated without spontaneous breathing, which means some patients will stay in the ICU only waiting for death to occur.

*“The problem is once they are intubated you do not extubate*, *that*’*s a legal issue*. *And brain dead is not always ruled out before they are intubated”*

(no 7)

It was mentioned that some postoperative patients take up space in ICU for long time because of surgery being done on patients with low chances of recovery or because of problems during surgery or anesthesia.

#### Fear of repercussions

Some patients with poor prognoses and without expected benefit from ICU care were said to still be admitted because of a fear of criticism. Doctors worried that they may end up in trouble for rejecting patients even if their decisions could be medically justifiable. The interviews did not fully reveal where the criticism would come from or what the punitive actions might be.

*“You cannot say no because of poor prognosis because of politics*. *They will come nail you if the patients end up dying and they will say it*’*s your fault*.*”*

(no 4)

*“Yes*, *you could end up in trouble*. *Like this case we were talking about today […] Everybody are talking about it*. *He might end up in kind of trouble*.*”*

(no 15)

#### VIP patients

“People of importance” were described as potentially getting easier access to ICU but at the time of the study it was thought that most of these people would be cared for in other hospitals. Government officials, people working in the health system or relatives were said to be favored. There were no experiences of patients admitted to ICU through financial means.

*“…we do get instructions to take important people to ICU*. *Personally I don*’*t like to work by titles*. *I don*’*t want to do something just because somebody is a big-shot*. *You should attend everybody equally*.*”*

(no 15)

*“Yes*, *important people they get special treatment*. *Administration contact us and tell us now you will receive this patient*, *they already prepared everything*, *they prepared where he should go*, *what should be done*, *everything*. *You are just told what to do*. *But now it*’*s not a problem for us anymore because now they all go to the new cardiac unit*.*”*

(no 4)

## Discussion

In a hospital in a low-income country, we have found that doctors perceive there to be difficulties with the identification of critically ill patients in the wards and a lack of structured triaging to the ICU.

The study respondents described that ward nurses do not always react when patients become critically ill and specialist doctors are too distant from the patients to identify critical illness. Triaging to ICU is affected by the lack of guidelines for admission, diverging ideas about ICU indications and contraindications and ward staff who do not consider ICU as an option for the care of their patients. Triaging to the ICU was further hampered by the lack of bed capacity in the ICU and non-medical factors such as a fear of repercussions. Admission of patients without expected benefit from ICU care was described as occurring due to fear of getting criticized. According to the respondents sometimes rejecting patient admission could lead to repercussions even if the rejection was medically justifiable, for example due to poor prognosis.

The failure of identification of the critically ill may be explained by a lack of critical care knowledge and communication barriers. A low level of knowledge about critical care among the staff has been pointed out previously in Tanzania and other sub-Saharan countries [34–36]. The majority of staff in Tanzanian hospitals have no formal training in critical care or triage (35–36) and there is a lack of formal prioritization systems for the critically ill (35). Implementation of emergency care and triage training has been shown to decrease patient mortality in LICs [37]. Communication between the ward teams and the ICU are done by specialist to specialist contact. As the medical system has several hierarchical levels of professionals, this requires communication between many health workers. This was described as creating a distance between the patient and the specialist that could result in delays in acting upon the identification of critical illness. Interprofessional hierarchies have been noted to have negative effects on communication and cooperation among healthcare staff [38] and to decrease staff motivation [39]. Communication difficulties were aggravated by specialists’ absence from the hospital. Dual practice is a known phenomenon in sub-Saharan Africa [40–43] mainly due to the need for income supplementation [40, 41]. Communication is further hampered by a lack of confidence, motivation and a low working morale among the nurses—similar to findings in other healthcare facilities in Tanzania [35, 44–47].

A reported barrier to effective ICU triage was a lack of explicit guidelines and routines. There was no consensus about admission criteria and no written guidelines for admission. This implies that decisions to refer patients to ICU, and to accept patients to ICU, is done on an ad-hoc basis and using implicit criteria that may not be uniformly understood or applied. The majority of patients admitted to the ICU were post-operative patients and patients who required mechanical ventilation, suggesting two possible implicit criteria. Postoperative patients have been considered a group who should be monitored closely and who may benefit from ICU care [48]. However, a recent study with data from 27 countries including LICs, showed no evidence of patient survival benefit after admission to ICU immediately postoperatively [49].

The ICU doctors expressed that the ICU is mainly for patients needing a respirator and the ward doctors believed that it would be futile to contact ICU about patients who could breathe without the need of mechanical ventilation. Favoring patients with a need for mechanical ventilation may prevent patients with circulatory shock or other critical illness from selection for ICU care until it is too late. It is considered as essential in intensive care medicine to admit patients before their condition advances beyond the point where recovery is impossible [2, 48, 50], and patients with shock may benefit from early intervention and ICU care [51–53]. If illness severity is the only implicit criterium used, there is a risk in intensive care medicine that patients with poor prognoses and very low chances of survival are selected above those who are less sick but for whom ICU care could be life-saving. This was described by the respondents in the study, both for post-operative patients who were known to have poor prognoses even before surgery and other patients with expected poor outcomes, and is also a challenge in other settings [5].

Underlying all the challenges of identifying and triaging the critically ill is the extreme imbalance of critical care needs and available resources. The patient selection process was perceived to be highly affected by the lack of space in the ICU which is consistent with previous findings in HICs [5, 9], whereby patients who get admitted in situations of limited number of beds, are more severely ill [7] and get admitted later in their illness [9]. In HICs, physicians respond to intensive care resource limitation by using the resources more efficiently [6, 7], however in these settings resources are not as limited as in a LIC like Tanzania. In this study, it was described that the chronic and severe scarcity of ICU beds deters staff from referring critically ill patients to the ICU resulting in patients with a need for intensive care remaining on the general wards while the e ICU was at times reported by study participants to have empty beds.

Admission decisions for ICU were not only based on potential benefit but could involve other non-medical factors such as fear of repercussions and orders to admit VIP patients. In a previous study at MNH punishment rather than rewards seemed to characterize the work environment and both doctors and nurses experienced a lack of care for their welfare by the employer [54]. Corruption is known to play a role in health care systems in sub-Saharan Africa [55], however the extent of VIP patients in our study seemed relatively low and was reported to be decreasing due to VIPs choosing care in other hospitals.

The study is unique in its focus on the under-researched area of triage and critical care in a low-resource setting that has potential for a global public health impact, using methods to explore implicit and convoluted processes in the identification and triage of patients for ICU in hospital. Centering interviews with health staff on real patient cases enabled reality-based discussions and made it easier to talk about sensitive and general issues.

An important methodological consideration is the positioning of the interviewer and the research team. Interviews conducted by a non-Tanzanian may have influenced respondents’ accounts, for example due to barriers inherent in cross-cultural communication, due to social desirability to answer “correctly” or due to altered openness of the respondents to an “outsider”. It is also possible that, due to the interviewer being an outsider, participants shared accounts more accurately reflecting reality than if the interviews had been conducted by a colleague from within the hospital.

Although the interviewer had no financial or other formal influence over respondents, there may have been inherent power imbalances that could have affected the interviews. This could have influenced respondents to try to give a good impression, or to exaggerate difficulties in order to gain sympathy and a potential future gain. The interviewer did not, however, get the impression that participants felt coerced to take part, nor hesitant to speak freely.

The positioning and value base of researchers influence analysis and interpretation in all qualitative studies. In our case, the lens through which data was collected and analyzed arrived from a position of wanting to understand a system and complex processes of care from the outside. The research team contained several doctors with high-income country backgrounds, and the team’s pre-conceived ideas about LIC healthcare and the management of critical illness likely influenced the interpretation of the findings. Respondents accounts could have been interpreted overly negatively but at the same time, the perspective and knowledge of healthcare from other settings may have led to insights that local colleagues could have missed.

The study had several limitations. The interviews were not recorded for logistical reasons and with the aim of increasing the possibility for respondents to speak openly, especially about some of the sensitive issues. This could have led to lost or misunderstood information or a premature interpretation by the interviewer. The interview settings were chosen by the respondents and several of the interviews were not done in total privacy. Although confidentiality was of concern, the study was pragmatic and the busy staff had to be interviewed close to their workplaces. The respondents were selected purposively for their recent involvement in the care of a critically ill patient and may not be representative of all doctors in MNH. As the study was done in a single hospital, transferability of the results to other settings should be done with caution.

We suggest that, in LIC settings where ICU beds are limited and the needs for critical care are great, the processes for identifying the critically ill and triaging of patients to ICU should be explicit. The processes should be widely known, accepted and used, and supported by the hospital and clinical leadership. Admission guidelines can be difficult to implement [12] and are not always followed by triaging ICU doctors [12, 15] and guidelines developed in high income countries can be challenging to apply in LICs [34, 56–58]. Despite these difficulties, explicit and written ICU admission guidelines in every ICU are seen as crucial by several intensive care professional societies [17, 48]. Local guidelines with clear criteria are valuable for identifying appropriate patients for ICU care in the current hospital context [2], decrease the impact of bias and prejudice and help limit the unnecessary use of resources in low-income countries [16]. As well as implementation of effective guidelines, critical care training of hospital staff, raising awareness about critical illness, and measures to improve communication, such as non-hierarchical triage systems, within hospitals could benefit the optimal identification and triage of critically ill patients. Further research should aim to study the implementation and impact of improved identification of critical illness and triaging to ICU and the impact on patient outcomes.

## Conclusion

Critically ill patients may not be identified in general wards in a Tanzanian university hospital and the triaging process for the admission of patients to intensive care is convoluted and not explicit. The findings indicate a potential for improved patient selection that could optimize the use of scarce ICU resources, leading to better patient outcomes.

## Supporting information

S1 Appendix Interview guide. (DOCX)

## References

[1] Fullerton JN, Perkins GD. Who to admit to intensive care? Clin Med (Lond). 2011;11(6):601–4. 22268319 10.7861/clinmedicine.11-6-601 PMC4952346

[2] Smith G, Nielsen M. ABC of intensive care. Criteria for admission. BMJ. 1999;318(7197):1544–7. 10356016 10.1136/bmj.318.7197.1544 PMC1115908

[3] Guidelines for intensive care unit admission, discharge, and triage. Task Force of the American College of Critical Care Medicine, Society of Critical Care Medicine. Crit Care Med. 1999;27(3):633–8. 10199547

[4] Attitudes of critical care medicine professionals concerning distribution of intensive care resources. The Society of Critical Care Medicine Ethics Committee. Crit Care Med. 1994;22(2):358–62. 8306698 10.1097/00003246-199402000-00031

[5] Vincent JL. European attitudes towards ethical problems in intensive care medicine: results of an ethical questionnaire. Intensive Care Med. 1990;16(4):256–64. 10.1007/bf01705162 2358559

[6] Singer DE, Carr PL, Mulley AG, Thibault GE. Rationing intensive care—physician responses to a resource shortage. N Engl J Med. 1983;309(19):1155–60. 10.1056/NEJM198311103091905 6413862

[7] Strauss MJ, LoGerfo JP, Yeltatzie JA, Temkin N, Hudson LD. Rationing of intensive care unit services. An everyday occurrence. JAMA. 1986;255(9):1143–6. 3945032

[8] Marshall MF, Schwenzer KJ, Orsina M, Fletcher JC, Durbin CG Jr. Influence of political power, medical provincialism, and economic incentives on the rationing of surgical intensive care unit beds. Crit Care Med. 1992;20(3):387–94. 10.1097/00003246-199203000-00016 1541100

[9] Sprung CL, Geber D, Eidelman LA, Baras M, Pizov R, Nimrod A, et al Evaluation of triage decisions for intensive care admission. Crit Care Med. 1999;27(6):1073–9. 10.1097/00003246-199906000-00021 10397207

[10] Garrouste-Orgeas M, Montuclard L, Timsit JF, Misset B, Christias M, Carlet J. Triaging patients to the ICU: a pilot study of factors influencing admission decisions and patient outcomes. Intensive Care Med. 2003;29(5):774–81. 10.1007/s00134-003-1709-z 12677368

[11] Sinuff T, Kahnamoui K, Cook DJ, Luce JM, Levy MM, Values E, et al Rationing critical care beds: a systematic review. Crit Care Med. 2004;32(7):1588–97. 10.1097/01.ccm.0000130175.38521.9f 15241106

[12] Azoulay E, Pochard F, Chevret S, Vinsonneau C, Garrouste M, Cohen Y, et al Compliance with triage to intensive care recommendations. Crit Care Med. 2001;29(11):2132–6. 10.1097/00003246-200111000-00014 11700409

[13] Truog RD, Brock DW, Cook DJ, Danis M, Luce JM, Rubenfeld GD, et al Rationing in the intensive care unit. Crit Care Med. 2006;34(4):958–63; quiz 71. 10.1097/01.CCM.0000206116.10417.D9 16484912

[14] Blanch L, Abillama FF, Amin P, Christian M, Joynt GM, Myburgh J, et al Triage decisions for ICU admission: Report from the Task Force of the World Federation of Societies of Intensive and Critical Care Medicine. J Crit Care. 2016;36:301–5. 10.1016/j.jcrc.2016.06.014 27387663

[15] Orsini J, Blaak C, Yeh A, Fonseca X, Helm T, Butala A, et al Triage of Patients Consulted for ICU Admission During Times of ICU-Bed Shortage. J Clin Med Res. 2014;6(6):463–8. 10.14740/jocmr1939w 25247021 PMC4169089

[16] van Zyl-Smit R, Burch V, Willcox P. The need for appropriate critical care service provision at non-tertiary hospitals in South Africa. S Afr Med J. 2007;97(4):268, 70, 72 17446951

[17] Curtis JR, Vincent JL. Ethics and end-of-life care for adults in the intensive care unit. Lancet. 2010;376(9749):1347–53. 10.1016/S0140-6736(10)60143-2 20934213

[18] Chen LM, Render M, Sales A, Kennedy EH, Wiitala W, Hofer TP. Intensive care unit admitting patterns in the Veterans Affairs health care system. Arch Intern Med. 2012;172(16):1220–6. 10.1001/archinternmed.2012.2606 22825806

[19] Cook D, Rocker G, Marshall J, Sjokvist P, Dodek P, Griffith L, et al Withdrawal of mechanical ventilation in anticipation of death in the intensive care unit. N Engl J Med. 2003;349(12):1123–32. 10.1056/NEJMoa030083 13679526

[20] Garland A, Connors AF. Physicians’ influence over decisions to forego life support. J Palliat Med. 2007;10(6):1298–305. 10.1089/jpm.2007.0061 18095808

[21] Cooper AB, Sibbald R, Scales DC, Rozmovits L, Sinuff T. Scarcity: the context of rationing in an Ontario ICU. Crit Care Med. 2013;41(6):1476–82. 10.1097/CCM.0b013e31827cab6a 23474676

[22] Guyatt G, Cook D, Weaver B, Rocker G, Dodek P, Sjokvist P, et al Influence of perceived functional and employment status on cardiopulmonary resuscitation directives. J Crit Care. 2003;18(3):133–41. 14595566 10.1016/j.jcrc.2003.08.001

[23] Adhikari NK, Fowler RA, Bhagwanjee S, Rubenfeld GD. Critical care and the global burden of critical illness in adults. Lancet. 2010;376(9749):1339–46. 10.1016/S0140-6736(10)60446-1 20934212 PMC7136988

[24] Kwizera A, Dunser M, Nakibuuka J. National intensive care unit bed capacity and ICU patient characteristics in a low income country. BMC research notes. 2012;5:475 10.1186/1756-0500-5-475 22937769 PMC3470976

[25] Manda-Taylor L, Mndolo S, Baker T. Critical care in Malawi: The ethics of beneficence and justice. Malawi medical journal: the journal of Medical Association of Malawi. 2017;29(3):268–71. 29872519 10.4314/mmj.v29i3.8 PMC5812001

[26] Data U. Country statistics United Republic of Tanzania 2017 [Available from: http://data.un.org/en/iso/tz.html.

[27] Baker T, Lugazia E, Eriksen J, Mwafongo V, Irestedt L, Konrad D. Emergency and critical care services in Tanzania: a survey of ten hospitals. BMC Health Serv Res. 2013;13:140 10.1186/1472-6963-13-140 23590288 PMC3639070

[28] Sawe HR, Mfinanga JA, Lidenge SJ, Mpondo BCT, Msangi S, Lugazia E, et al Disease patterns and clinical outcomes of patients admitted in intensive care units of tertiary referral hospitals of Tanzania. BMC international health and human rights. 2014;14:26 10.1186/1472-698X-14-26 25245028 PMC4204389

[29] Baker T, Schell CO, Lugazia E, Blixt J, Mulungu M, Castegren M, et al Vital Signs Directed Therapy: Improving Care in an Intensive Care Unit in a Low-Income Country. PLoS One. 2015;10(12):e0144801 10.1371/journal.pone.0144801 26693728 PMC4687915

[30] Baker T, Blixt J, Lugazia E, Schell CO, Mulungu M, Milton A, et al Single Deranged Physiologic Parameters Are Associated With Mortality in a Low-Income Country. Critical care medicine. 2015;43(10):2171–9. 10.1097/CCM.0000000000001194 26154933

[31] Halpern NA, Pastores SM, Greenstein RJ. Critical care medicine in the United States 1985–2000: An analysis of bed numbers, use, and costs. Critical care medicine. 2004;32(6):1254–9. 10.1097/01.ccm.0000128577.31689.4c 15187502

[32] Kruisselbrink R, Kwizera A, Crowther M, Fox-Robichaud A, O’Shea T, Nakibuuka J, et al Modified Early Warning Score (MEWS) Identifies Critical Illness among Ward Patients in a Resource Restricted Setting in Kampala, Uganda: A Prospective Observational Study. PloS one. 2016;11(3):e0151408 10.1371/journal.pone.0151408 26986466 PMC4795640

[33] Hsieh HF, Shannon SE. Three approaches to qualitative content analysis. Qual Health Res. 2005;15(9):1277–88. 10.1177/1049732305276687 16204405

[34] Baker T. Critical care in low-income countries. Trop Med Int Health. 2009;14(2):143–8. 10.1111/j.1365-3156.2008.02202.x 19207174

[35] Baker T L E, Eriksen J, Mwafongo V, Irestedt L & Konrad D Emergency and critical care services in Tanzania: a survery of ten hospitals. BMC Health Services Research 2013;13(140). 10.1186/1472-6963-13-140 PMC3639070 23590288

[36] Aloyce R, Leshabari S, Brysiewicz P. Assessment of knowledge and skills of triage amongst nurses working in the emergency centres in Dar es Salaam, Tanzania. African Journal of Emergency Medicine. 2014;4(1):14–8.

[37] Molyneux E, Ahmad S, Robertson A. Improved triage and emergency care for children reduces inpatient mortality in a resource-constrained setting. Bull World Health Organ. 2006;84(4):314–9. 10.2471/blt.04.019505 16628305 PMC2627321

[38] Rice K, Zwarenstein M, Conn LG, Kenaszchuk C, Russell A, Reeves S. An intervention to improve interprofessional collaboration and communications: a comparative qualitative study. J Interprof Care. 2010;24(4):350–61. 10.3109/13561820903550713 20540614

[39] Mathauer I, Imhoff I. Health worker motivation in Africa: the role of non-financial incentives and human resource management tools. Hum Resour Health. 2006;4:24 10.1186/1478-4491-4-24 16939644 PMC1592506

[40] Abera GG, Alemayehu YK, Herrin J. Public-on-private dual practice among physicians in public hospitals of Tigray National Regional State, North Ethiopia: perspectives of physicians, patients and managers. BMC Health Serv Res. 2017;17(1):713 10.1186/s12913-017-2701-6 29126453 PMC5681802

[41] Russo G, McPake B, Fronteira I, Ferrinho P. Negotiating markets for health: an exploration of physicians’ engagement in dual practice in three African capital cities. Health Policy Plan. 2014;29(6):774–83. 10.1093/heapol/czt071 24077880 PMC4153303

[42] Ashmore J, Gilson L. Conceptualizing the impacts of dual practice on the retention of public sector specialists—evidence from South Africa. Hum Resour Health. 2015;13:3 10.1186/1478-4491-13-3 25600159 PMC4320565

[43] Paina L, Bennett S, Ssengooba F, Peters DH. Advancing the application of systems thinking in health: exploring dual practice and its management in Kampala, Uganda. Health Res Policy Syst. 2014;12:41 10.1186/1478-4505-12-41 25134522 PMC4142472

[44] Manongi RN, Marchant TC, Bygbjerg IC. Improving motivation among primary health care workers in Tanzania: a health worker perspective. Hum Resour Health. 2006;4:6 10.1186/1478-4491-4-6 16522213 PMC1450300

[45] Mkoka DA, Mahiti GR, Kiwara A, Mwangu M, Goicolea I, Hurtig AK. "Once the government employs you, it forgets you": Health workers’ and managers’ perspectives on factors influencing working conditions for provision of maternal health care services in a rural district of Tanzania. Hum Resour Health. 2015;13:77 10.1186/s12960-015-0076-5 26369663 PMC4570215

[46] Songstad NG, Rekdal OB, Massay DA, Blystad A. Perceived unfairness in working conditions: the case of public health services in Tanzania. BMC Health Serv Res. 2011;11:34 10.1186/1472-6963-11-34 21314985 PMC3045290

[47] Khamis K, Njau B. Health care worker’s perception about the quality of health care at the outpatient department in Mwananyamala Hospital in Dar es Salaam, Tanzania. Tanzania Journal of Health Research. 2016;18(1).

[48] Nates JL, Nunnally M, Kleinpell R, Blosser S, Goldner J, Birriel B, et al ICU Admission, Discharge, and Triage Guidelines: A Framework to Enhance Clinical Operations, Development of Institutional Policies, and Further Research. Crit Care Med. 2016;44(8):1553–602. 10.1097/CCM.0000000000001856 27428118

[49] Kahan BC, Koulenti D, Arvaniti K, Beavis V, Campbell D, Chan M, et al Critical care admission following elective surgery was not associated with survival benefit: prospective analysis of data from 27 countries. Intensive Care Med. 2017;43(7):971–9. 10.1007/s00134-016-4633-8 28439646

[50] Workgroup VD. Ethical Considerations for Decision Making Regarding Allocation of Mechanical Ventilators during a Severe Influenza Pandemic or Other Public Health Emergency. Atlanta, GA: Centers for Disease Control and Prevention, 2011.

[51] Sebat F, Musthafa AA, Johnson D, Kramer AA, Shoffner D, Eliason M, et al Effect of a rapid response system for patients in shock on time to treatment and mortality during 5 years. Crit Care Med. 2007;35(11):2568–75. 10.1097/01.CCM.0000287593.54658.89 17901831

[52] Cardoso LT, Grion CM, Matsuo T, Anami EH, Kauss IA, Seko L, et al Impact of delayed admission to intensive care units on mortality of critically ill patients: a cohort study. Crit Care. 2011;15(1):R28 10.1186/cc9975 21244671 PMC3222064

[53] Brown RM, Semler MW. Fluid Management in Sepsis. J Intensive Care Med. 2018:885066618784861. 10.1177/0885066618784861 PMC6532631 29986619

[54] Leshabari MT, Muhondwa EP, Mwangu MA, Mbembati NA. Motivation of health care workers in Tanzania: a case study of Muhimbili National Hospital. East Afr J Public Health. 2008;5(1):32–7. 18669121 10.4314/eajph.v5i1.38974

[55] Mostert S, Njuguna F, Olbara G, Sindano S, Sitaresmi MN, Supriyadi E, et al Corruption in health-care systems and its effect on cancer care in Africa. Lancet Oncol. 2015;16(8):e394–404. 10.1016/S1470-2045(15)00163-1 26248847

[56] Dunser MW, Baelani I, Ganbold L. A review and analysis of intensive care medicine in the least developed countries. Crit Care Med. 2006;34(4):1234–42. 10.1097/01.CCM.0000208360.70835.87 16484925

[57] Dunser MW, Festic E, Dondorp A, Kissoon N, Ganbat T, Kwizera A, et al Recommendations for sepsis management in resource-limited settings. Intensive Care Med. 2012;38(4):557–74. 10.1007/s00134-012-2468-5 22349419 PMC3307996

[58] Wheeler I, Price C, Sitch A, Banda P, Kellett J, Nyirenda M, et al Early warning scores generated in developed healthcare settings are not sufficient at predicting early mortality in Blantyre, Malawi: a prospective cohort study. PLoS One. 2013;8(3):e59830 10.1371/journal.pone.0059830 23555796 PMC3612104

