# Supplementary material for: Referral and admission to intensive care: A qualitative study of doctors’ practices in a Tanzanian university hospital
Source: PLoS One. 2019 Oct 29;14(10):e0224355. doi: 10.1371/journal.pone.0224355 (PMC6818781; doi:10.1371/journal.pone.0224355)
Supplement: S1 Appendix — (DOCX) [file pone.0224355.s001.docx]

S1 Appendix. Interview guide.

**Referral and admission to intensive care: a qualitative study of doctors’ practices in a Tanzanian university hospital**

(1) What do you know about official ICU referral and admission criteria in MNH?

- Are there any official criteria for ICU admission at MNH? If yes, please describe them.
- In your experience, are they being used? If yes, how?

(2) What were your experiences of ICU referral and admission process and decision making in the specific patient case?

- Describe the referral procedure in the specific case
- Describe the admission procedure in the specific case

(3) How do you experience communication during the process of referral and admission to the ICU in general?

- How is communication done between doctors during ICU referrals?
- Who in the ICU should be contacted, and how should this be done?
- Could you describe obstacles you have had to refer/admit patients to ICU?

(4) What do you perceive to be the clinical indications for ICU care?

- Which patient categories may be suitable for ICU care at MNH?
- Could you describe if you are aware of any contraindications to ICU care at MNH?

(5) What are your perceptions of the quality of care of critically ill patients in ICU and the general wards?

- What do you think about the quality of care of critically ill patients in ICU and in the wards
- What do you perceive to be the obstacles to providing good quality of care in the ICU?

(6) What do you think about the influence of non-medical factors on ICU admission?

- Do you think patients sometimes are admitted to ICU because of money or importance?
